# Pharmacological Akt and JNK Kinase Inhibitors 10-DEBC and SP600125 Potentiate Anti-Glioblastoma Effect of Menadione and Ascorbic Acid Combination in Human U251 Glioblastoma Cells

**DOI:** 10.3390/biomedicines11102652

**Published:** 2023-09-27

**Authors:** Ana Despotović, Kristina Janjetović, Nevena Zogović, Gordana Tovilović-Kovačević

**Affiliations:** 1 Department of Neurophysiology, Institute for Biological Research “Siniša Stanković”—National Institute of the Republic of Serbia, University of Belgrade, Bulevar despota Stefana 142, 11000 Belgrade, Serbia; ana.despotovic@ibiss.bg.ac.rs (A.D.); kristina.janjetovic@ibiss.bg.ac.rs (K.J.); 2 Department of Biochemistry, Institute for Biological Research “Siniša Stanković”—National Institute of the Republic of Serbia, University of Belgrade, Bulevar despota Stefana 142, 11000 Belgrade, Serbia

**Keywords:** glioblastoma therapy, menadione and ascorbic acid, Akt inhibitor 10-DEBC, JNK inhibitor SP600125, oxidative stress, autophagy

## Abstract

Glioblastoma multiforme (GBM) is the most lethal primary brain tumor in adults, characterized by a highly invasive nature and therapy resistance. Combination of menadione and ascorbic acid (AA+MD) exerts strong ROS-mediated anti-GBM activity in vitro. The objective of this study was to improve AA+MD anti-GBM potential by modulating the activity of Akt and c-Jun N-terminal kinase (JNK), molecules with an important role in GBM development. The effects of Akt and JNK modulation on AA+MD toxicity in U251 human glioblastoma cells were assessed by cell viability assays, flow cytometry, RNA interference and plasmid overexpression, and immunoblot analysis. The AA+MD induced severe oxidative stress, an early increase in Akt phosphorylation followed by its strong inhibition, persistent JNK activation, and U251 cell death. Small molecule Akt kinase inhibitor 10-DEBC enhanced, while pharmacological and genetic Akt activation decreased, AA+MD-induced toxicity. The U251 cell death potentiation by 10-DEBC correlated with an increase in the combination-induced autophagic flux and was abolished by genetic autophagy silencing. Additionally, pharmacological JNK inhibitor SP600125 augmented combination toxicity toward U251 cells, an effect linked with increased ROS accumulation. These results indicate that small Akt and JNK kinase inhibitors significantly enhance AA+MD anti-GBM effects by autophagy potentiation and amplifying deleterious ROS levels.

## 1. Introduction

Glioblastoma multiforme (GBM) is a grade IV astrocytoma originating from neuroepithelial glial cells and represents the most lethal primary brain tumor in adults. Despite multimodal therapy comprised of surgery, postoperative radiotherapy, and chemotherapy with alkylating agent temozolomide, the median overall survival of GBM patients is ~14.6 months [1,2,3]. This remarkably poor prognosis is a result of the highly invasive GBM nature and development of radio-and chemo-resistance due mostly to numerous genetic alterations and deregulated signaling pathways [2,3]. The most common genetic alterations identified in GBM patients by The Cancer Genome Atlas project are related to the overactivation of the receptor tyrosine kinase (RTK)/Ras/phosphatidylinositol 3-kinase (PI3K) pathway (88% of all cases), gained by the mutation/amplification of RTKs and PI3K, or loss of function of PTEN (phosphatase and tensin homolog), a phosphatase that counteracts action of PI3K. Activated PI3K further stimulates the protein kinase B (Akt)/mammalian target of rapamycin (mTOR) pathway and promotes GBM cell growth, proliferation, and survival through hyperactivation of multiple effector molecules [3]. In addition to growth support, elevated activity of the PI3K/Akt/mTOR axis, acquired in response to conventional therapy, provides GBM with significant chemo- and radio-resistance [4]. The activation of mitogen-activated protein kinases (MAPKs) cascade member c-Jun N-terminal kinase (JNK) was also reported in GBM and correlated with high histological grade and the poor prognosis of GBM patients [5,6]. Moreover, the elevated JNK activity is related to increased GBM proliferation and maintenance of glioma stem cell properties, contributing to the high self-renewal capacity of GBM [6,7]. In addition, simultaneous activation of PI3K/Akt and JNK pathways was described in several tumors with mutated PTEN, including GBM [8].

Targeting the members of signaling pathways altered in GBM using small molecule inhibitors has gained notable attention as a strategy to suppress GBM progression and overcome its resistance to conventional therapy. Numerous studies in cell lines and animal GBM models show potent anti-GBM effects of PI3K/Akt/mTOR and MAPK/JNK inhibitors, exerted mainly through proliferative suppression, apoptosis induction, autophagy impairment, or by restricting the tumor-initiating potential of GBM cells [9,10,11,12,13,14,15,16]. Moreover, several ongoing clinical trials are assessing the potential of PI3K, Akt, and mTOR inhibitors in combating GBM, either as a monotherapy or in combination with conventional therapeutics [17,18]. While selective Akt inhibitors, such as perifosine, AZD5363, and MK-2206, have been extensively studied, JNK inhibitors have not entered clinical trials as potential anticancer therapeutics so far primarily due to lack of specificity for different JNK isoforms [12,15]. However, systemic toxicity and the feedback activation of proliferative pathways remain the main challenges in cancer/GBM monotherapy with small molecule kinase inhibitors [19]. To overcome this challenge and enhance anticancer efficacy, the most recommendable approach is the combination of kinase inhibitors with radiotherapy, chemotherapy, or other targeting agents.

Menadione (MD, vitamin K3, 2-methyl-1,4-naphtoquinone) alone, and in combination with ascorbic acid (AA), has been shown to possess significant anticancer activity against different cancer types, including GBM, in vitro and in vivo [20,21,22,23,24,25]. The anticancer effect of both MD and the AA+MD combination is mediated by oxidative stress, namely, cellular quinone reductases metabolize MD to hydroquinone through semiquinone intermediate, producing a superoxide anion. AA, on the other hand, drives one-electron redox-cycling of MD, generating excessive concentrations of superoxide anion radicals and hydrogen peroxide in the cell. Through the elevation of reactive oxygen species (ROS), MD exerts both antiproliferative and cytotoxic actions, whereas AA+MD-generated excessive oxidative stress induces cancer cell death [20,21,25,26,27,28]. Types of cell death induced by MD and its combination include either conventional (apoptosis, necrosis, autophagy) or alternative (autoschizis) modalities [20,27,28,29]. In addition to its pro-oxidant activity, AA exerts significant anticancer potential through diverse mechanisms. They include restriction of cancers’ metastatic potential by inhibition of hyaluronidase, an enzyme responsible for degradation of extracellular matrix and cancer cell dissemination; epigenetic recovery in the expression of tumor suppressor genes through the restored TET-regulated DNA demethylation process; as well as suppression of hypoxia-inducible factor 1 (HIF1), a transcription factor that allows adaptation of cancer cells to hypoxic environment [30,31]. It should be emphasized that pharmacologically achievable concentrations of AA and MD selectively target cancer cells with a minor effect on the redox state and viability of non-transformed cells and tissues [23,24,25]. Moreover, AA+MD (also known as Apatone^®^) has gained an orphan drug status for the treatment of stage III and IV bladder cancer and has shown promising results in patients with advanced prostate cancer who had failed standard therapy [32,33].

Our previous study revealed that AA+MD exerted significant anti-GBM effects through induction of AMPK/mTORC1/ULK1-dependent cytotoxic autophagy and subsequent U251 GBM cell death, and MD stimulated autophagy without causing significant toxicity towards U251 cells [28]. In this study, we aimed to improve the efficacy of MD and AA+MD against GBM cells via pharmacological modulation using small molecule inhibitors of two commonly deregulated signaling pathways in GBM—PI3K/Akt and MAPK/JNK. The results revealed that 10-DEBC augmented AA+MD and triggered MD anti-GBM effects through autophagy potentiation and elevation of ROS levels, while SP-mediated JNK suppression enhanced anti-GBM action of both MD and AA+MD through oxidative stress exacerbation.

## 2. Materials and Methods

### 2.1. Cell Culture and Treatments

The U251 cell line was obtained from the European Collection of Authenticated Cell Cultures (ECACC, Salisbury, UK), while the human fetal lung fibroblast cell line MRC5 was obtained from the American Type Culture Collection (ATCC, Manassas, VI, USA). The cultures were maintained at 37 °C in a humidified atmosphere with 5% CO_2_ in the RPMI 1640 cell culture medium (Gibco, Life Technologies, Waltham, MA, USA) supplemented with 5% (U251) or 10% (MRC5) fetal bovine serum (FBS), and 1% antibiotic-antimycotic solution (10^5^ U/L penicillin, 100 mg/L streptomycin, 0.25 mg/L amphotericin B) (all from Capricorn Scientific, Ebsdorfergrund, Germany). The cells were incubated in 96-well flat-bottom plates (1 × 10^4^ cells/well) for the cell viability assays, 6-well plates (2.5 × 10^5^ cells/well) for the flow cytometry analysis, and 60 mm Petri dishes (1 × 10^6^ cells) for the immunoblotting. After seeding, U251 or MRC5 cells were rested for 24 h and then treated as described in figure legends. 10-DEBC and SP were added to the cells 30 min before AA and/or MD administration. AA and MD were added to the cells simultaneously.

10-DEBC hydrochloride was purchased from Tocris Bioscience (Bristol, UK), insulin was from Novartis (Basel, Switzerland), while all other chemicals used in this study were from Sigma-Aldrich (St. Louis, MO, USA).

### 2.2. Cell Viability Assessment

Crystal violet (CV) staining of adherent, viable cells, and mitochondrial-dependent reduction of 3-(4,5-dimethylthiazol-2-yl)-2,5-diphenyl-tetrazolium bromide (MTT) (Sigma-Aldrich, St. Louis, MO, USA) to formazan as an indicator of mitochondrial dehydrogenase activity, were used to assess the viability of U251 cells. Before CV and MTT tests, the cells were washed with phosphate-buffered saline (PBS) to remove dead cells and to circumvent MTT and AA interaction. For the CV assay, the adherent cells were fixed with methanol (15 min) and incubated with the CV (1%) at room temperature (15 min). The excess dye was vigorously washed off with tap water. The remaining dye was dissolved in acetic acid (33%). For the MTT assay, cells were incubated with an MTT solution (0.5 mg/mL) for 1 h. To dissolve formed formazan salts, dimethyl sulfoxide was added to the wells. The absorbance of colored solutions was measured at 570 nm (Synergy H1, Biotek, Winooski, VT, USA). The results were shown as % of viability relative to untreated control cells (100% viable).

### 2.3. Light Microscopy

A Bio-Rad ZOE fluorescent cell imager (Bio-Rad Laboratories, Hercules, CA, USA) was used for the bright field live images of human U251 glioblastoma cells.

### 2.4. Analysis of Apoptosis and Necrosis

The type of cell death was determined by double staining cells with annexin V-FITC/PI (BD Biosciences, Heidelberg, Germany), according to the manufacturer’s recommendations. The green (FITC; FL1) and red (PI; FL2) fluorescence was measured using a BD FACSAria III flow cytometer (BD Biosciences, San Jose, CA, USA) to determine the number of viable (Ann^−^/PI^−^), early apoptotic (Ann^+^/PI^−^), and late apoptotic/necrotic cells (Ann^+^/PI^+^).

### 2.5. Caspase Activation

To determine caspase activation, detached cells were stained with pan-caspase inhibitor ApoStat (R&D Systems, Minneapolis, MN, USA) according to the manufacturer’s recommendations. The increase in green fluorescence (FITC; FL1) as a measure of caspase activity was determined using a BD FACSAria III flow cytometer. The results were expressed as the fold change in the mean fluorescence intensity relative to the value obtained in untreated cells, which was arbitrarily set to 1.

### 2.6. Measurement of ROS Production

The total intracellular concentration of ROS was analyzed by measuring the fluorescence of cells stained with redox-sensitive dye dihydrorhodamine 123 (DHR) according to the manufacturer’s recommendations (Thermo Fisher Scientific, Waltham, MA, USA). The mean intensity of green fluorescence (FITC; FL1) corresponding to total ROS concentration was determined using a BD FACSAria III flow cytometer. The results were presented as the fold change relative to the mean fluorescence value of the untreated cells, which was arbitrarily set to 1.

### 2.7. Intracellular Detection of Acidic Vesicles and Autophagic Vacuoles

Acridine orange staining was used to visualize the acidic vesicles. After treatment indicated in figure legends, cells were washed, stained with acridine orange (1 μM) for 15 min at 37 °C, and analyzed under a Zeiss Axiovert fluorescent microscope (Zeiss, Oberkochen, Germany). Autophagolysosomes and lysosomes appeared as red fluorescent cytoplasmic vesicles, while nuclei were stained green.

### 2.8. Immunoblotting

Immunoblot analysis was performed exactly as previously described [28]. Nitrocellulose membranes (Bio-Rad, Hercules, CA, USA) were incubated with rabbit anti-human antibodies against total-Akt, phospho-Akt (Ser473), total-JNK, phospho-JNK (Thr183/Tyr185), microtubule-associated protein 1 light chain 3B (LC3B), β-actin (Cell Signaling Technology, Danvers, MA, USA), total-Bcl2, phospho-Bcl2 (Ser70) (Santa Cruz Biotechnology, Dallas, TX, USA), and mouse anti-human antibody against p62/sequestosome 1 (Novus Biologicals, Littleton, CO, USA), as primary antibodies. Peroxidase-conjugated goat anti-rabbit IgG (Cell Signaling Technology, Dallas, TX, USA) and goat anti-mouse IgG (Southern Biotech, Birmingham, AL, USA) were used as secondary antibodies. Specific protein bands were visualized by enhanced chemiluminescence using the Amersham reagent (Amersham Pharmacia Biotech, Piscataway, NJ, USA) on the ChemiDoc Imaging System (BioRad Laboratories, Hercules, CA, USA). The signal intensity was quantified by densitometry using ImageJ software (version 1.51j8, National Institutes of Health, Bethesda, MA, USA). The ratio of phospho/total protein or LC3-II and p62 relative to actin was calculated. The results were presented relative to the signal intensity of the untreated control, which was arbitrarily set to 1.

### 2.9. RNA Interference and Plasmid Overexpression

Knockdown of gene expression was conducted using small interfering RNA (siRNA) targeting Human LC3 and JNK, as well as scrambled control siRNA (Santa Cruz Biotechnology). Expression of Akt was augmented through transient transfection of constitutively active myristoylated Akt (Myr-Akt) construct, kindly provided by Professor Marc Peters-Golden (University of Michigan, Ann Arbor, MI, USA). Transfection of U251 cells with siRNA (100 nM) and plasmids (1 ng/µL, Myr-Akt and control construct) using Lipofectamine 2000 (2.8 µg/mL) (Thermo Fisher Scientific, Waltham, MA, USA) was performed according to the manufacturer’s recommendations. Cells were allowed to grow for 24 h following transfection and treated as described in figure legends.

### 2.10. Statistical Analysis

The statistical significance of the differences was analyzed by *t*-test or one-way ANOVA followed by the Student–Newman–Keuls test. A *p*-value of less than 0.05 was considered statistically significant.

## 3. Results

### 3.1. The Combination of AA and MD Induces Necrotic Cell Death in U251 Cells Mediated by Oxidative Stress

**Figure 1:**
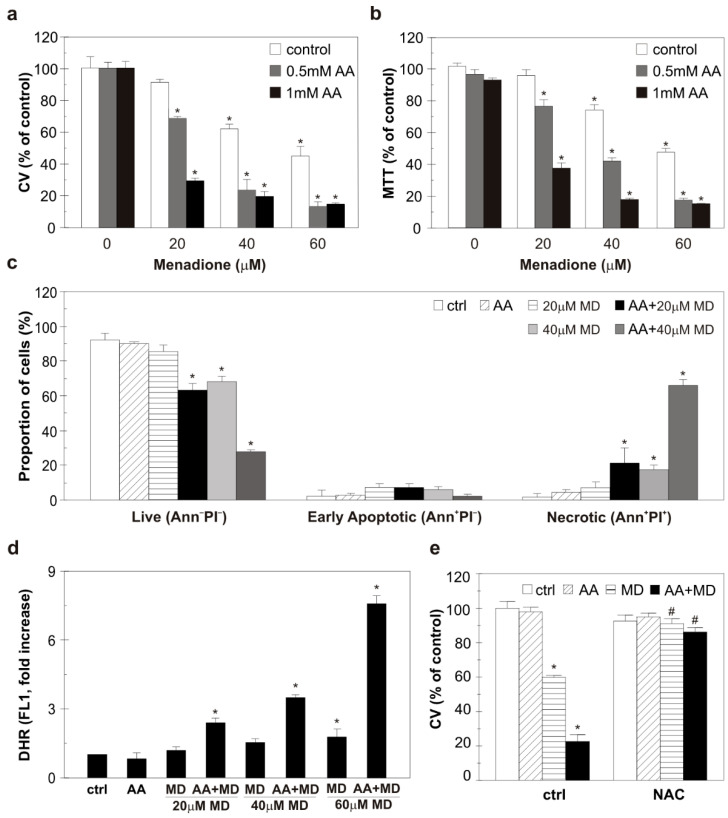
The combination of AA and MD induces oxidative stress and necrosis in U251 cells. U251 cells were incubated with different concentrations of AA (0.5–1 mM), MD (20–60 µM), and their appropriate combinations (AA+MD) for 16 h (**a**,**b**), 6 h (**c**), or 2 h (**d**). Alternatively, cells were treated with 1 mM AA and MD 40 µM, and their combination in the absence or presence of N-acetyl cysteine (2 mM, NAC) for 16 h (**e**). Cell viability was assessed using crystal violet (**a**,**e**) and MTT (**b**) tests. Fluorescence intensity of the cells stained with AnnexinV^-^FITC/PI (**c**) and DHR (**d**), indicating the presence of apoptotic/necrotic cells and intracellular ROS production, respectively, was measured using flow cytometry. The data are presented as mean ± SD values of triplicates (**a**–**e**) from one representative of three independent experiments. * *p* < 0.05 compared to control, untreated cells (ctrl), # *p* < 0.05 compared to cells treated with MD or AA+MD.

U251 human glioblastoma cells, an in vitro model of GBM, were treated with different concentrations of AA (0.5 mM, 1 mM), MD (20–60 µM), and their combinations for 16 h. The U251 cell line was selected for its PTEN and p53 mutation status. AA applied alone did not affect cell viability, while MD in higher concentrations (40 and 60 µM; MD_high_) and the combination AA+MD significantly reduced the number of viable adherent cells and mitochondrial dehydrogenase activity in a dose-dependent manner, as measured by CV (Figure 1a) and MTT (Figure 1b) assays, respectively. In line with previous results, flow cytometry of cells stained with Annexin V-FITC/PI revealed that single AA (1 mM) and MD (20 μM; MD_low_) treatment did not affect the number of live/dead cells [28]. The higher MD dose (40 μM) and AA+MD combination in a dose-dependent manner elevated the proportion of necrotic cells with the ruptured membrane (Ann^+^PI^+^), while the number of early apoptotic cells (Ann^+^/PI^−^) was not altered by either treatment (Figure 1c). According to the well-known ability of MD and AA+MD to induce oxidative stress, a flow cytometric analysis of DHR-stained cells revealed an early (2 h), dose-dependent increase in intracellular ROS levels in MD-treated cells and, to a much higher extent, in combination-treated cells (Figure 1d) [21,25,28]. The presence of well-established antioxidant NAC vigorously opposed the AA+MD and MD_high_ (40 μM) cytotoxic potential toward U251 cells (Figure 1e), suggesting a central role of oxidative stress in AA+MD-triggered cell death. These findings confirm that the AA+MD combination induces necrotic death of U251 cells via oxidative stress.

### 3.2. AA+MD Combination and MD Modulate Akt and JNK Activity through Oxidative Stress in U251 Cells

**Figure 2:**
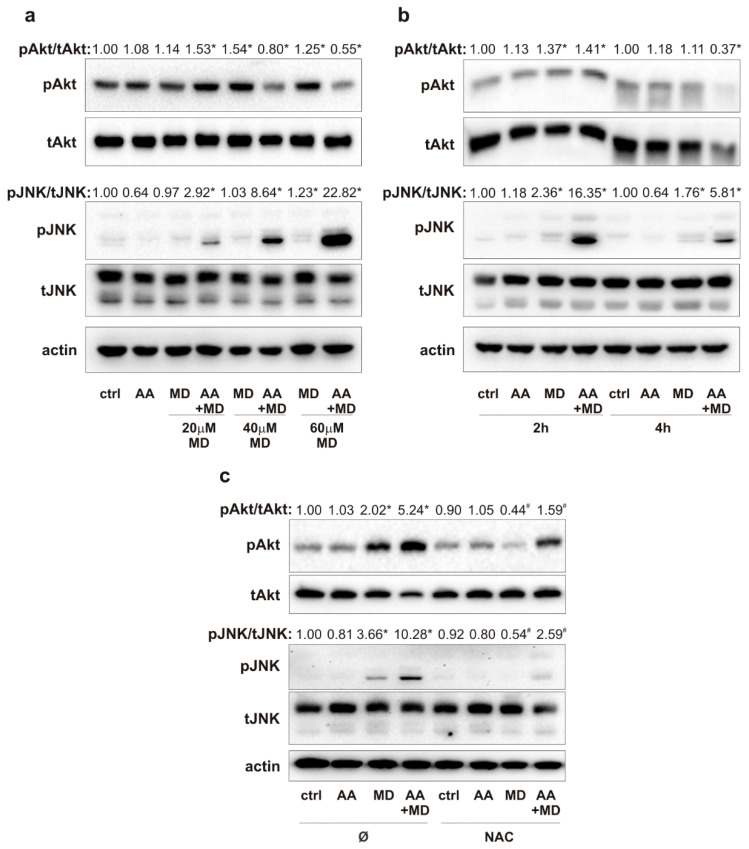
MD and combination activate Akt and JNK via ROS generation. U251 cells were incubated with AA (1 mM) and different concentrations of MD (20–60 µM), and their appropriate combinations (AA+MD), for 1 h (**a**) and 2 h and 4 h (**b**) or cells were pre-treated with N-acetyl cysteine (NAC; 2 mM) for 30 min and exposed to AA (1 mM) and MD (20 µM) for 2 h (**c**). The levels of phospho-Akt (pAkt), total-Akt (tAkt), phospho-JNK (pJNK), total-JNK (tJNK), and actin as a loading control were determined by immunoblotting and quantified using densitometry. The images of representative immunoblots are shown, and data are presented as mean values of one out of three independent experiments. * *p* < 0.05 compared to control, untreated cells (ctrl), # *p* < 0.05 compared to cells treated with MD or AA+MD.

To evaluate the effect of MD and AA+MD on key signaling molecules commonly altered in GBM, we performed immunoblot analysis of Akt and JNK activity in U251 cells treated with increasing MD concentrations (20–60 µM), a single AA concentration (1 mM), and their combinations (Figure 2a). Treatment with MD and AA+MD_low_ (20 µM) activated, while AA+MD_high_ (40 and 60 µM) inhibited, the activity of the main pro-survival kinase Akt after 1 h of treatment. Concomitantly, MD at the highest dose, and especially AA+MD, increased phosphorylation of JNK in a dose-dependent manner (Figure 2a). The concentrations of 20 µM MD and 1 mM of AA were chosen for further experiments since their combination decreased cell viability by 70% after 16 h treatment and was associated with mild changes in Akt and JNK activity. Time-course immunoblot analysis of Akt activity showed a transient increase in Akt phosphorylation induced by MD and AA+MD after 2 h, followed by a decline to control levels in MD and a strong inhibition in AA+MD after prolonged (4 h) incubation (Figure 2b). At the same time, MD induced mild, while the combination induced strong and persistent, JNK activation (Figure 2b). Next, we explored the impact of MD- and AA+MD-generated oxidative stress on changes in Akt and JNK activities, since ROS can affect the function of both Akt and JNK signaling pathways [34,35]. Therefore, U251 cells were exposed to N-acetyl cysteine (NAC), a well-known antioxidant agent, and treated with AA, MD, and their combination for 2 h. Immunoblot analysis demonstrated that diminishing oxidative stress with NAC counteracted MD- and AA+MD-related activation of Akt and JNK signaling pathways (Figure 2c). Our results suggest that MD increases the phosphorylation of Akt and JNK, while prolonged AA+MD treatment results in strong inhibition of Akt and robust and persistent activation of the JNK signaling pathway. Furthermore, ROS are involved in the early induction of Akt and JNK signaling pathways by MD and AA+MD combination.

### 3.3. Small Molecule Inhibitors of Akt and JNK Enhance Anti-GBM Effect of Combination and Trigger MD Cytotoxicity through Oxidative Stress Potentiation

**Figure 3:**
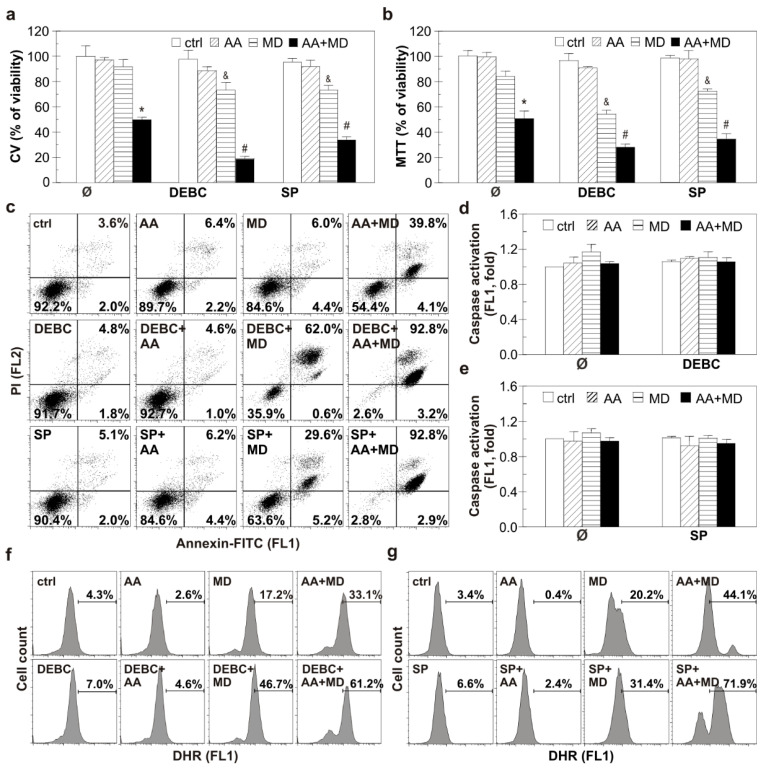
Selective inhibition of Akt with DEBC and JNK with SP potentiate apoptosis-independent AA+MD-triggered U251 cell death and induces MD cytotoxicity. Untransfected U251 cells (**a**–**g**) were treated with AA (1 mM), MD (20 µM), and their combination (AA+MD) in the presence or absence of selective Akt inhibitor 10-DEBC (10 µM) or selective JNK inhibitor SP (10 µM) for 16 h (**a**,**b**), 6 h (**c**–**e**), or 2 h (**f**,**g**). Cell viability was assessed by crystal violet (CV) and MTT assays (**a**,**b**). U251 cells were stained with AnnexinV-FITC/PI (**c**), ApoStat (**d**,**e**), and DHR (**f**,**g**); fluorescence intensity indicating the presence of apoptotic/necrotic cells (**c**), caspase activation (**d**,**e**), and intracellular ROS production (**f**,**g**) was measured using flow cytometry. The data are presented as mean ± SD values of triplicates (**a**,**b**,**d**,**e**), representative dot plots (**c**), or histograms (**f**,**g**) from one representative of three independent experiments. * *p* < 0.05 compared to control, untreated cells (ctrl), # *p* < 0.05 compared to control cells treated with AA+MD, & *p* < 0.05 compared to control cells treated with MD.

The ability of AA+MD and MD to activate Akt and JNK in U251 cells through ROS generation prompted us to determine the potential of Akt and JNK small molecule inhibitors to modulate the anti-GBM effect of MD and AA+MD. Treatment of U251 cells with 10-DEBC, ATP-competitive Akt inhibitor, significantly enhanced toxicity of combination and aroused toxicity of MD alone, as shown by CV and MTT assays (Figure 3a,b) [36]. Similar results were obtained with another Akt inhibitor—perifosine (Figure S1). For further experiments, 10-DEBC was chosen since it was more efficient in U251 cell death induction. Pharmacological inhibition of JNK with the selective ATP-competitive inhibitor SP promoted AA+MD-triggered cell death and increased sensitivity of U251 cells to MD, as shown by CV and MTT assays (Figure 3a,b) [15]. Previous results were confirmed with siRNA-mediated downregulation of JNK. Namely, genetic inhibition of JNK decreased the number of viable cells in both MD and AA+MD treatments compared to control cells treated with MD and AA+MD, as the CV test showed (Figure S2). Bright-field light microscopy showed strong cytoplasmic vacuolization and a significant decrease in cell density in AA+MD-treated U251 cells (Figure S3). Following pre-treatment with 10-DEBC or SP, the number of remaining viable cells in AA+MD treatment rapidly declined, while cells with robust cellular vacuolization escalated. Interestingly, both 10-DEBC and SP in MD-treated U251 cells triggered morphological changes that resembled those observed in cell cultures treated with the combination, i.e., strong cellular vacuolization and decay in cell number. No changes in cell morphology were noticed in cells treated with 10-DEBC or SP alone (Figure S3). Considering the important role of both Akt and JNK in apoptosis regulation, we next investigated the capacity of 10-DEBC and SP to initiate apoptosis in MD- and AA+MD-treated cells [37,38]. Flow cytometry of Annexin V-FITC/PI-labeled cells showed that both 10-DEBC and SP increased the number of Ann^+^/PI^+^ cells with a damaged membrane compared to the corresponding events in MD (otherwise non-toxic) and AA+MD, without affecting the early apoptotic Ann^+^/PI^−^ events (Figure 3c). In addition, flow cytometry of cells stained with pan-caspase inhibitor ApoStat showed that both 10-DEBC (Figure 3d) and SP (Figure 3e) failed to induce caspase activation in the presence of AA+MD or MD. Overall, the obtained results imply that apoptosis was neither involved in the cytotoxicity of AA+MD nor activated by 10-DEBC and SP.

To reveal mechanisms underlying the cytotoxic potential of investigated small molecule inhibitors, we tested their potential to interfere with MD- and AA+MD-induced ROS accumulation. Flow cytometric analysis of cells labeled with DHR showed that both 10-DEBC and SP increased the intracellular ROS levels in cells treated with MD and AA+MD compared to MD and AA+MD alone, respectively (Figure 3f,g). These results suggest that exacerbation of cell death by Akt and JNK inhibition in our experimental setting is presumably related to oxidative stress.

### 3.4. 10-DEBC Enhances Anti-GBM Effect of MD and AA+MD by Potentiating Cytotoxic Autophagy

**Figure 4:**
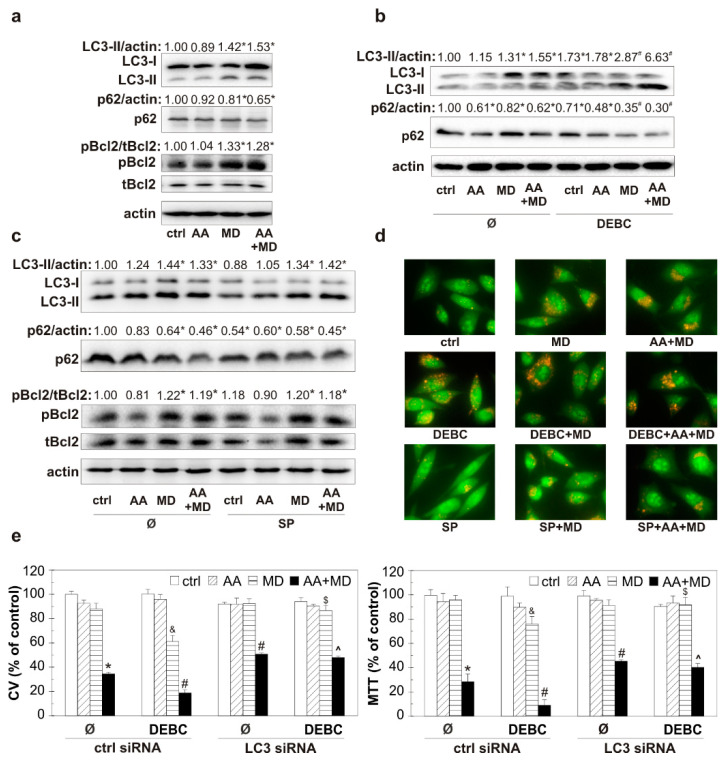
The opposite effect of 10-DEBC and SP on MD- and AA+MD-triggered autophagy in U251 cells. Untransfected U251 cells (**a**–**d**) and cells transfected with control or LC3 siRNA (**e**) were incubated with AA (1 mM), MD (20 μM), and AA+MD in the absence or presence of Akt inhibitor 10-DEBC (10 μM) (**b**,**d**) and JNK inhibitor SP (10 μM) (**c**,**d**) for 1 h (**a**–**d**) or 16 h (**e**). Cell viability was evaluated by crystal violet (CV) and MTT (**e**) assays. The levels of LC3-II, p62, phospho-Bcl2 (pBcl2), total-Bcl2 (tBcl2), and actin as a loading control were determined by immunoblotting and quantified using densitometry (**a**–**c**). The presence of acridine-orange-stained acidic vesicular organelles was determined using fluorescent microscopy (**d**). The data are presented as mean ± SD values of triplicates from one representative out of three independent experiments (**e**), as representative immunoblots (**a**–**c**) or pictures (**d**) from one out of three independent experiments. * *p* < 0.05 compared to control, untreated cells (ctrl), # *p* < 0.05 compared to control cells treated with AA+MD, & *p* < 0.05 compared to control cells treated with MD, $ *p* < 0.05 compared to control cells treated with 10-DEBC+MD, ^ *p* < 0.05 compared to control cells treated with 10-DEBC+AA+MD.

Having in mind the ability of MD and AA+MD to promote autophagy and the involvement of Akt and JNK in autophagy regulation, we next examined the ability of small molecule kinase inhibitors 10-DEBC and SP to affect the key autophagy-related proteins and subsequently well-defined cytotoxic autophagy in our system [28,39,40]. Furthermore, considering that JNK kinase can promote autophagy by phosphorylating Bcl2, we wanted to monitor the impact of our treatments on this molecule, which plays an important role in two fundamental cell death pathways. Consistently with our previous study, immunoblot analysis showed elevated levels of autophagosome-associated LC3-II isoform and p62 degradation in MD- and AA+MD-treated cells (Figure 4a) [28]. Moreover, we have herein, for the first time, shown that Bcl2 phosphorylation occurred simultaneously with the engagement of autophagy in MD and AA+MD treatments of U251 cells (Figure 4a). Pre-treatment with 10-DEBC induced an additional rise in LC3-II levels compared with the corresponding treatments without 10-DEBC (Figure 4b). Accordingly, the levels of specific autophagic substrate and cargo-related p62 protein were significantly lower in 10-DEBC+MD- and 10-DEBC+combination-treated cells than in cells exposed to MD and combination alone (Figure 4b). These results suggest further potentiation of autophagy in 10-DEBC-treated cells. Interestingly, immunoblot analysis showed that SP did not affect MD- and AA+MD-induced changes in the levels of autophagy markers LC3-II and p62 (Figure 4c), suggesting JNK-independent autophagy induction under our experimental conditions. Accordingly, MD- and AA+MD-triggered phosphorylation of Bcl-2, a Beclin1-interacting autophagy inhibitor, was not affected by the JNK inhibitor SP (Figure 4c). In line with the previous findings were results obtained after the analysis of acridine-orange-loaded cells using fluorescent microscopy (Figure 4d). The increased number of intracytoplasmic autolysosome-like acidic vesicles detected in MD and AA+MD treatment was further raised in the presence of small kinase inhibitor 10-DEBC. SP had no additional impact on the number of acidic vesicular organelles observed in U251 cells challenged with MD and AA+MD. To examine the role of potentiated autophagy in 10-DEBC-induced toxicity, autophagy was silenced by LC3 siRNA. A genetic knockdown of autophagy partially restored the viability of AA+MD-treated cells and abolished 10-DEBC-associated toxicity enhancement in MD and AA+MD treatments, as CV and MTT tests showed (Figure 4e). Collectively, these data suggest that 10-DEBC enhances the toxicity of MD alone and AA+MD through the potentiation of cytotoxic autophagy, while SP-mediated exacerbation of U251 cell death in our experimental setting is not related to the potentiation of autophagy.

### 3.5. Pharmacological and Genetic Activation of Akt Diminishes ROS Generation, Autophagy Promotion, and GBM Cell Death Induced by AA+MD

**Figure 5:**
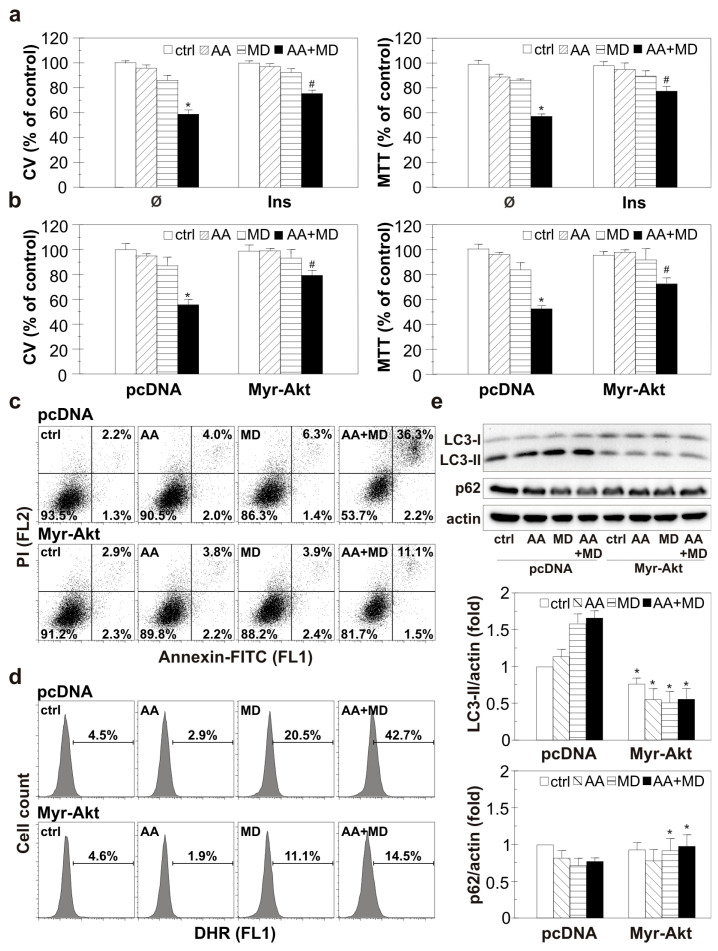
Pharmacological and genetic Akt activation diminishes ROS accumulation, autophagy promotion, and U251 cell death induced by AA+MD. Untransfected U251 cells pre-treated with insulin (40 mU/mL, Ins) (**a**) or U251 cells transfected with myristoylated Akt (Myr-Akt) and control PCDNA plasmid vector (**b**–**e**) were incubated with AA (1 mM), MD (20 µM), and AA+MD for 16 h (**a**,**b**), 6 h (**c**), 2 h (**d**), or 1 h (**e**). The number of viable, adherent cells and cells with active mitochondria was evaluated by CV and MTT assays, respectively (**a**,**b**). The presence of apoptotic/necrotic cells was assessed by flow cytometry of AnnexinV-FITC/PI-stained cells (**c**). The fluorescence intensity of DHR-stained U251 cells indicating intracellular ROS production (**d**) was measured using flow cytometry. The levels of LC3-II, p62, and actin as a loading control were determined by immunoblotting and quantified using densitometry (**e**). The data are presented as mean ± SD values of triplicates from one representative of three independent experiments (**a**,**b**), or as dot plots (**c**), histograms (**d**), or as representative immunoblots (**e**) from one out of three independent experiments. * *p* < 0.05 compared to control, untreated cells (**a**,**b**) or pcDNA cells (**e**), # *p* < 0.05 compared to control or PCDNA cells treated with AA+MD.

To evaluate the molecular events that link Akt and AA+MD-induced GBM cell death, we used pharmacological and genetic overactivation of the Akt signaling pathway and exposed cells to AA and MD for a prolonged period (16 h). Pharmacological activation of Akt using a well-known PI3K/Akt inducer, insulin, partially recovered the viability of AA+MD-treated cells, as CV and MTT assays showed (Figure 5a) [37]. In accordance, overexpression of Akt with constitutively active myristoylated Akt (myr-Akt) construct partially prevented cell death triggered by AA+MD, restoring to some extent the number of viable adherent and cells with active mitochondria, as shown by CV and MTT assays (Figure 5b) [41]. A flow cytometric analysis of cells stained with Annexin V-FITC/PI demonstrated AA+MD-caused shift of live Ann^−^/PI^−^ cells to Ann^+^/PI^+^ cells with a ruptured membrane, while genetic overexpression of Akt reduced the number of necrotic-like Ann^+^/PI^+^ cells in AA+MD treatment (Figure 5c). These results suggest that Akt overactivation prevents cell membrane damage induced by AA+MD. Moreover, MD- and combination-induced increase in total ROS was diminished in U251 cells with genetically overexpressed Akt compared to cells transfected with a control plasmid, as demonstrated by flow cytometric analysis of DHR-stained cells (Figure 5d). Finally, genetic overexpression of Akt completely abolished the elevation of LC3-II levels and p62 degradation observed in MD- and combination-treated control cells (Figure 5e). Altogether, these results confirm the role of Akt in opposing the damaging effect of AA+MD, probably due to interference with increased ROS and autophagy levels.

### 3.6. The Investigated Combinations Do Not Exert Toxicity toward Human Lung Fibroblasts

**Figure 6:**
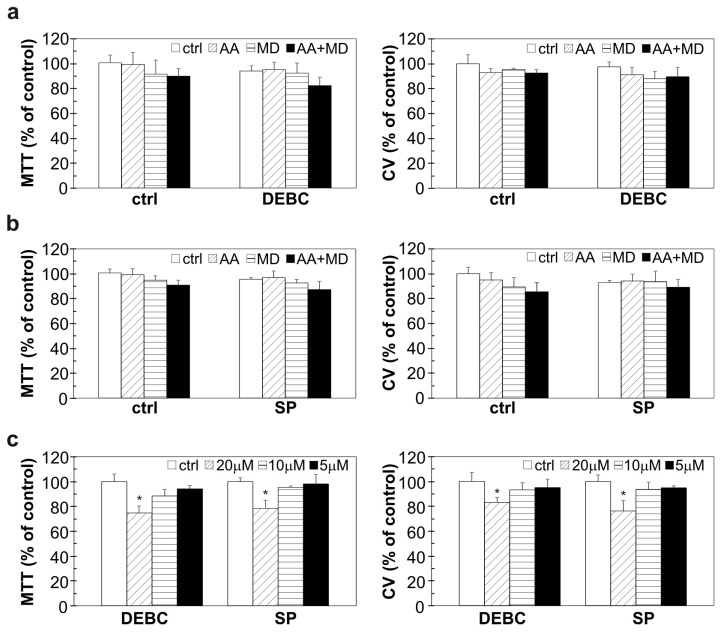
The influence of 10-DEBC, SP, AA, and MD on the viability of human lung fibroblast cells. MRC5 cells were pre-treated with 10-DEBC (10 µM) and SP (10 µM) for 30 min and then exposed to AA (1 mM), MD (20 µM), and AA+MD (1 mM + 20 µM) (**a**,**b**) or treated with different concentrations of 10-DEBC and SP (5–20 µM) (**c**) for 24 h. Cell viability was assessed by MTT and CV assay. The data are presented as mean values ± SD of triplicates from one representative of two independent experiments. * *p* < 0.05 vs control cells (ctrl).

To evaluate the selectivity of investigated combinations towards GBM cells, we conducted experiments on human lung fibroblasts MRC5. CV and MTT tests showed that the viability of MRC5 cells was not significantly affected by the applied treatments (Figure 6a,b). The highest concentration of 10-DEBC and SP (20 µM) had minimal effect on the viability of healthy cells (Figure 6c). These findings indicate selectivity of applied treatments towards GBM cells and the absence of significant toxicity of 10-DEBC and SP toward healthy cells.

## 4. Discussion

The present study demonstrates the capacity of small kinase Akt and JNK inhibitors, 10-DEBC and SP, to potentiate the toxicity of MD and AA+MD, well-known oxidative stress generators, towards GBM cells in vitro. The mechanisms underlying the enhancing effect of 10-DEBC include an Akt attenuation-mediated increase in deleterious ROS and cytotoxic autophagy levels, resulting in augmented cellular demise. SP-related JNK inhibition exacerbates AA+MD and triggers MD’s anti-GBM effect through enhancement of ROS levels and U251 cell death, independently of autophagy modulation.

Potentiation of ROS-mediated U251 cell death by Akt and JNK attenuation observed here is in accordance with the ability of other Akt inhibitors MK-2206 (allosteric), perifosine (alkyl-phospholipid), Akt inhibitor IV (ATP-competitive), and JNK inhibitors AS602801 and SP (ATP-competitive) to increase irradiation-, temozolomide-, gefitinib-, and vincristine-induced death of various GBM cell lines in vitro [9,10,11,16,42,43]. ATP-competitive inhibitors 10-DEBC and SP in our study potentiate necrosis, a finding partially complying with the study of Huang et al., who reported perifosine-triggered increase of both apoptosis and necrosis in irradiated C6 GBM cells [9]. In contrast, in other studies, Akt and JNK inhibitors mainly delayed growth or induced apoptosis in GBM cells in response to radio- and chemotherapy [9,10,11,43]. The necrosis potentiation in this study is probably due to the specific cytotoxic mechanism of applied stressors, as quinone compounds deplete levels of apoptosis-required ATP and cause rapid caspase-independent cell death, especially in the case of AA+MD combination [27,28]. In addition, the absence of caspase activation may be related to the highly oxidative environment or p53 mutational status of the U251 cell line since both ROS and mutated p53 have been shown to interfere with caspase-3 activity and processing [44,45]. Further, ROS-mediated membrane damage, suggested in our study by the rapid increase in the number of Ann^+^PI^+^ cells, has been shown to strongly support necrotic over the other types of cell demise [46]. Thus, it seems that 10-DEBC and SP accelerate or enhance death-related processes already activated by MD and AA+MD in U251 cells. The fact that pan-Akt and pan-JNK inhibition augments MD/AA+MD toxicity by increased production of ROS and/or potentiation of autophagy, processes already linked with deleterious effects of MD and AA+MD in GBM cells, supports this assumption [24,28]. However, it should be highlighted that the biological effect of various Akt and JNK inhibitors depends highly on their pharmacological properties and selectivity profiles, as well as on the mechanisms of inhibition of Akt/JNK and their downstream molecules [15,47].

Both Akt and JNK have been involved in GBM tumorigenesis, invasiveness, and resistance to antineoplastic agents and radiation and enhanced activity of these protein kinases in response to conventional therapy is reported in established and primary GBM cell lines [48,49,50,51,52,53]. Accordingly, we observed ROS-mediated phosphorylation of these signaling pathways in MD and AA+MD treatments in U251 cells. Of note, both MD and AA+MD have been shown to induce ROS-triggered DNA damage, a hallmark of conventional anti-GBM therapy [54,55]. The finding on MD-induced Akt and JNK activation is in line with previous studies conducted in various cancer cell lines, while transient Akt phosphorylation, followed by its strong inhibition, and robust JNK activation in AA+MD-treated GBM cells, is demonstrated here for the first time [56,57]. Increased Akt and JNK activity may be a consequence of oxidative stress-mediated loss of function of their negative regulators, whereas vigorously elevated ROS after prolonged AA+MD treatment could force a close interaction of Akt with phosphatase 2A and its subsequent dephosphorylation [34,35,58]. Activation of endogenous Akt activity in response to chemotherapy counteracts the anti-GBM effects of chemotherapeutic agents through multiple downstream Akt targets, such as apoptosis-related Bcl2 family proteins and transcription factors responsible for transcription of antioxidant enzymes (HIF-1, FoxO3, NF-kB, Nrf2) [48]. Our data are consistent with studies showing that Akt opposes oxidative stress-induced toxicity by an increase in cellular antioxidative capacity, while the absence of Akt interaction with the apoptotic pathway, observed herein, can be at least partly explained by the stimuli-related necrosis induction [35,59]. The former assumption is corroborated by the results showing that selective Akt inhibitor 10-DEBC increased AA+MD and triggered MD toxicity by ROS accumulation, while Akt overexpression decreased ROS accumulation in MD and combination treatment and rescued U251 cells from AA+MD-induced death. However, the exact mechanisms underlying 10-DEBC-mediated disturbance in antioxidant defense should be further elucidated. The role of JNK in oxidative stress-induced cancer cell death is more complex, as it has been shown that ROS-mediated sustained high JNK activation induces apoptotic and/or necrotic cell death, while low levels of JNK activity support cancer cell survival through DNA repair stimulation and increased antioxidant production [60,61]. In addition, JNK activation correlates with death induction in GBM cells exposed to resveratrol nanoparticles [53,62]. We have detected both low levels of JNK activation in MD (without causing death) and a robust increase in JNK activity in combination (lethal necrotic stimulus). SP potentiated GBM cell death and enhanced ROS accumulation in both treatments. The former finding was also confirmed after genetic suppression of JNK activity. The observed increase in ROS after JNK inhibition partially complies with the studies showing JNK-mediated suppression of ROS accumulation in serum-deprived schwannoma cells and UV-irradiated fibroblasts [63,64]. Contrary to the protective role of JNK in MD-treated cells, implied by our data and the data of others showing SP-mediated sensitization of U87 GBM cells and hepatocytes to death from temozolomide and MD, the function of JNK in combined treatment is less clear [16,65]. Sustained activation could suggest JNK involvement in necrosis induction, but its pharmacological and genetic inhibition augments U251 cell death. It could be proposed that early JNK activation mediates survival response and that simultaneous activation of both Akt and JNK is necessary for JNK to accomplish its pro-survival function, as already suggested by Lamb et al. [66]. Akt inactivation in the later phases of combined treatment or early JNK inhibition, by SP or JNK siRNA, may disrupt survival balance and switch it toward cell death in AA+MD treatment. In addition, given the complex interplay between PI3K/Akt and JNK pathways in cancer and the proposed role of JNK in priming Akt activity in GBM response to therapy, the possible mutual interaction between these pathways in our experimental conditions should not be excluded [8,16].

Autophagy induction by MD and AA+MD observed here is in accordance with the results of our previous study, showing ROS-triggered AMPK/mTOR/ULK1-dependent autophagy in MD- and AA+MD-treated cells [28]. Pre-treatment with SP did not affect autophagy levels, while 10-DEBC significantly enhanced the number of autophagic-like vesicles, as well as the levels of autophagosome-associated LC3-II protein and p62 degradation in MD and combination treatment. These results argue against JNK involvement in autophagy regulation in our experimental setting and suggest an Akt inhibition-dependent increase in autophagy flux in 10-DEBC-treated U251 cells. The presumable role of Akt inhibition in autophagy initiation was further corroborated by the blunted autophagy response in cells with overexpressed Akt. Given the role of Akt in the mTOR-mediated suppression of autophagy initiation, it may be assumed that Akt inhibition by 10-DEBC increased input for autophagosome formation, while ROS generation in MD and AA+MD provided additional impulse for autophagy progression, resulting in autophagy potentiation in MD- and combination-treated U251 cells [39]. Although evidence of increased autophagy in the presence of other Akt inhibitors in GBM cells already exists, the role of induced autophagy in GBM is highly controversial with data corroborating both protective and detrimental effects of therapy-induced autophagy [11,13,67,68,69]. Here, genetic autophagy silencing partially abolished the pro-death effects of 10-DEBC in GBM cells confirming the cytotoxic nature of potentiated autophagy. This result implies that increased pro-autophagic input through inhibition of the PI3K/Akt pathway could make GBM cells more susceptible to ROS-generating lethal stimuli. In line with this assumption are data showing that otherwise non-toxic ROS-generating MD becomes harmful in the presence of an Akt inhibitor. The sensitization to oxidative insult by autophagy enhancers could be achieved by selective autophagic targeting of ROS scavenger catalase or degradation of selective autophagy receptors p62/SQSTM1 and BNIP3 [70].

The small molecule kinase inhibitors 10-DEBC and SP applied alone in our study did not affect U251 cell viability but sensitized U251 GBM cells to the sub-toxic concentration of MD. Simultaneous treatment resulted in a moderate decrease in U251 cell viability, suggesting an additive rather than synergistic interaction between these molecules. Regardless of the nature of their interaction, our results suggest that co-treatment of GBM cells with MD and Akt or JNK inhibitors may be proposed as a potential anti-GBM strategy. Furthermore, the previous hypothesis has a good basis in the fact that small molecules and MD can cross the blood−brain barrier [71,72]. In addition to a significant number of studies showing therapeutic values of JNK inhibitor SP, our study highlights 10-DEBC as an Akt inhibitor that deserves future detailed research regarding its anticancer potential [15].

The treatment-induced necrosis observed in our study, potentiated in the presence of the small molecule inhibitors, may represent a desirable therapy-induced GBM cell death pattern bearing in mind that cells with defective apoptotic pathways confer a resistant phenotype to GBM [73]. Although our results were obtained using a single cell line and require further confirmation, they imply that in addition to the well-established role of the PI3K/Akt pathway in GBM therapy, the strategy of JNK inhibition may be employed as a tool for enhancing GBM response to therapy. The significant anticancer potential of AA+MD recently described in other GBM cell lines (U87, GS9L) and in vivo GBM models [23,24], together with ongoing clinical studies of PI3K/Akt/mTOR inhibitors, make further investigation of the anticancer potential of the combinations proposed herein reasonable. In addition, although no anti-GBM effect of AA alone or in combination with small molecule inhibitors was observed in the current study, substantial anticancer potential of pharmacological AA doses (millimolar range) in vivo through mechanisms independent of those investigated herein cannot be overlooked [30,31]. Considering the contribution of PI3K/Akt and JNK pathways to GBM progression and chemoresistance to a wide range of therapeutics, these results may help to further define potential therapeutic targets for effective GBM therapy [3,5].

## Data Availability

Data will be made available on request.

## Supplementary Materials

The following supporting information can be downloaded at: https://www.mdpi.com/article/10.3390/biomedicines11102652/s1, Figure S1: Akt inhibitor perifosine potentiates AA+MD-triggered U251 cell death and induces MD cytotoxicity; Figure S2: JNK downregulation with siRNA potentiates AA+MD-triggered U251 cell death and induces MD cytotoxicity; Figure S3: Morphological changes in the U251 cells treated with 10-DEBC, SP, MD, and combination; Figure S4: Myristoylated Akt transfection efficiency.

## References

[1] Stupp R., Hegi M.E., Mason W.P., van den Bent M.J., Taphoorn M.J., Janzer R.C., Ludwin S.K., Allgeier A., Fisher B., Belanger K. (2009). Effects of radiotherapy with concomitant and adjuvant temozolomide versus radiotherapy alone on survival in glioblastoma in a randomised phase III study: 5-year analysis of the EORTC-NCIC trial. Lancet Oncol..

[2] Wen P.Y., Weller M., Lee E.Q., Alexander B.M., Barnholtz-Sloan J.S., Barthel F.P., Batchelor T.T., Bindra R.S., Chang S.M., Chiocca E.A. (2020). Glioblastoma in adults: A Society for Neuro-Oncology (SNO) and European Society of Neuro-Oncology (EANO) consensus review on current management and future directions. Neuro. Oncol..

[3] Liu H., Qiu W., Sun T., Wang L., Du C., Hu Y., Liu W., Feng F., Chen Y., Sun H. (2022). Therapeutic strategies of glioblastoma (GBM): The current advances in the molecular targets and bioactive small molecule compounds. Acta Pharm. Sin. B.

[4] Colardo M., Segatto M., Di Bartolomeo S. (2021). Targeting RTK-PI3K-mTOR Axis in Gliomas: An Update. Int. J. Mol. Sci..

[5] de los Reyes Corrales T., Losada-Pérez M., Casas-Tintó S. (2021). JNK Pathway in CNS Pathologies. Int. J. Mol. Sci..

[6] Bubici C., Papa S. (2014). JNK signalling in cancer: In need of new, smarter therapeutic targets. Br. J. Pharmacol..

[7] Kitanaka C., Sato A., Okada M. (2013). JNK Signaling in the Control of the Tumor-Initiating Capacity Associated with Cancer Stem Cells. Genes Cancer.

[8] Zhao H.F., Wang J., To S.S.T. (2015). The phosphatidylinositol 3-kinase/Akt and c-Jun N-terminal kinase signaling in cancer: Alliance or contradiction? (Review). Int. J. Oncol..

[9] Huang K., Zhao W., Wang X., Qiu Y., Liu Z., Chen R., Liu W., Liu B. (2021). Akt Inhibition Enhanced the Growth Inhibition Effects of Low-Dose Heavy-Ion Radiation via the PI3K/Akt/p53 Signaling Pathway in C6 Glioblastoma Cells. Front. Oncol..

[10] Narayan R.S., Fedrigo C.A., Brands E., Dik R., Stalpers L.J.A., Baumert B.G., Slotman B.J., Westerman B.A., Peters G.J., Sminia P. (2017). The allosteric AKT inhibitor MK2206 shows a synergistic interaction with chemotherapy and radiotherapy in glioblastoma spheroid cultures. BMC Cancer.

[11] Cheng Y., Zhang Y., Zhang L., Ren X., Huber-Keener K.J., Liu X., Zhou L., Liao J., Keihack H., Yan L. (2012). MK-2206, a novel allosteric inhibitor of Akt, synergizes with gefitinib against malignant glioma via modulating both autophagy and apoptosis. Mol. Cancer Ther..

[12] Song M., Bode A.M., Dong Z., Lee M.H. (2019). AKT as a Therapeutic Target for Cancer. Cancer Res..

[13] Ríos-Marco P., Ríos A., Jiménez-López J.M., Carrasco M.P., Marco C. (2015). Cholesterol homeostasis and autophagic flux in perifosine-treated human hepatoblastoma HepG2 and glioblastoma U-87 MG cell lines. Biochem. Pharmacol..

[14] Matsuda K.I., Sato A., Okada M., Shibuya K., Seino S., Suzuki K., Watanabe E., Narita Y., Shibui S., Kayama T. (2012). Targeting JNK for therapeutic depletion of stem-like glioblastoma cells. Sci. Rep..

[15] Wu Q., Wu W., Jacevic V., Franca T.C.C., Wang X., Kuca K. (2020). Selective inhibitors for JNK signalling: A potential targeted therapy in cancer. J. Enzym. Inhib. Med. Chem..

[16] Vo V.A., Lee J.-W., Lee H.J., Chun W., Lim S.Y., Kim S.-S. (2014). Inhibition of JNK potentiates temozolomide-induced cytotoxicity in U87MG glioblastoma cells via suppression of Akt phosphorylation. Anticancer Res..

[17] ClinicalTrials.gov. GBM Pers. Trial. https://clinicaltrials.gov/ct2/show/NCT05432518.

[18] ClinicalTrials.gov A Trial Ipatasertib Comb. With Atezolizumab. https://classic.clinicaltrials.gov/ct2/show/NCT03673787.

[19] Kaley T.J., Panageas K.S., Mellinghoff I.K., Nolan C., Gavrilovic I.T., DeAngelis L.M., Abrey L.E., Holland E.C., Lassman A.B. (2019). Phase II trial of an AKT inhibitor (perifosine) for recurrent glioblastoma. J. Neurooncol..

[20] Lee M.H., Cho Y., Kim D.H., Woo H.J., Yang J.Y., Kwon H.J., Yeon M.J., Park M., Kim S.-H., Moon C. (2016). Menadione induces G2/M arrest in gastric cancer cells by down-regulation of CDC25C and proteasome mediated degradation of CDK1 and cyclin B1. Am. J. Transl. Res..

[21] Bonilla-Porras A.R., Jimenez-Del-Rio M., Velez-Pardo C. (2011). Vitamin K3 and vitamin C alone or in combination induced apoptosis in leukemia cells by a similar oxidative stress signalling mechanism. Cancer Cell Int..

[22] Sajadimajd S., Yazdanparast R. (2015). Differential behaviors of trastuzumab-sensitive and -resistant SKBR3 cells treated with menadione reveal the involvement of Notch1/Akt/FOXO1 signaling elements. Mol. Cell. Biochem..

[23] Sumiyoshi A., Shibata S., Zhelev Z., Miller T., Lazarova D., Zlateva G., Aoki I., Bakalova R. (2021). Pharmacological Strategy for Selective Targeting of Glioblastoma by Redox-active Combination Drug—Comparison with the Chemotherapeutic Standard-of-care Temozolomide. Anticancer Res..

[24] Sumiyoshi A., Shibata S., Zhelev Z., Miller T., Lazarova D., Aoki I., Obata T., Higashi T., Bakalova R. (2022). Targeting Glioblastoma via Selective Alteration of Mitochondrial Redox State. Cancers.

[25] Beck R., Verrax J., Gonze T., Zappone M., Pedrosa R.C., Taper H., Feron O., Calderon P.B. (2009). Hsp90 cleavage by an oxidative stress leads to its client proteins degradation and cancer cell death. Biochem. Pharmacol..

[26] Wiraswati H.L., Hangen E., Sanz A.B., Lam N.V., Reinhardt C., Sauvat A., Mogha A., Ortiz A., Kroemer G., Modjtahedi N. (2016). Apoptosis inducing factor (AIF) mediates lethal redox stress induced by menadione. Oncotarget.

[27] Majiene D., Kuseliauskyte J., Stimbirys A., Jekabsone A. (2019). Comparison of the Effect of Native 1,4-Naphthoquinones Plumbagin, Menadione, and Lawsone on Viability, Redox Status, and Mitochondrial Functions of C6 Glioblastoma Cells. Nutrients.

[28] Despotović A., Mirčić A., Misirlić-Denčić S., Harhaji-Trajković L., Trajković V., Zogović N., Tovilović-Kovačević G. (2022). Combination of Ascorbic Acid and Menadione Induces Cytotoxic Autophagy in Human Glioblastoma Cells. Oxid. Med. Cell. Longev..

[29] Gilloteaux J., Jamison J.M., Neal D., Summers J.L. (2014). Synergistic antitumor cytotoxic actions of ascorbate and menadione on human prostate (DU145) cancer cells in vitro: Nucleus and other injuries preceding cell death by autoschizis. Ultrastruct. Pathol..

[30] Reang J., Sharma P.C., Thakur V.K., Majeed J. (2021). Understanding the Therapeutic Potential of Ascorbic Acid in the Battle to Overcome Cancer. Biomolecules.

[31] Roa F.J., Peña E., Gatica M., Escobar-Acuña K., Saavedra P., Maldonado M., Cuevas M.E., Moraga-Cid G., Rivas C.I., Muñoz-Montesino C. (2020). Therapeutic Use of Vitamin C in Cancer: Physiological Considerations. Front. Pharmacol..

[32] Tareen B., Summers J.L., Jamison J.M., Neal D.R., McGuire K., Gerson L., Diokno A. (2008). A 12 week, open label, phase I/IIa study using apatone for the treatment of prostate cancer patients who have failed standard therapy. Int. J. Med. Sci..

[33] U.S. Food and Drug Administration (2007). Search Orphan Drug Des. Approv. https://www.accessdata.fda.gov/scripts/opdlisting/oopd/.

[34] Zhang J., Wang X., Vikash V., Ye Q., Wu D., Liu Y., Dong W. (2016). ROS and ROS-Mediated Cellular Signaling. Oxid. Med. Cell. Longev..

[35] Koundouros N., Poulogiannis G. (2018). Phosphoinositide 3-Kinase/Akt Signaling and Redox Metabolism in Cancer. Front. Oncol..

[36] Lee D.G., Kim H.J., Lee Y., Kim J.H., Hwang Y., Ha J., Ryoo S. (2022). 10-DEBC Hydrochloride as a Promising New Agent against Infection of Mycobacterium abscessus. Int. J. Mol. Sci..

[37] Nitulescu G.M., Van De Venter M., Nitulescu G., Ungurianu A., Juzenas P., Peng Q., Olaru O.T., Grǎdinaru D., Tsatsakis A., Tsoukalas D. (2018). The Akt pathway in oncology therapy and beyond (Review). Int. J. Oncol..

[38] Redza-Dutordoir M., Averill-Bates D.A. (2016). Activation of apoptosis signalling pathways by reactive oxygen species. Biochim. Biophys. Acta.

[39] Kma L., Baruah T.J. (2022). The interplay of ROS and the PI3K/Akt pathway in autophagy regulation. Biotechnol. Appl. Biochem..

[40] Zhou Y.Y., Li Y., Jiang W.Q., Zhou L.F. (2015). MAPK/JNK signalling: A potential autophagy regulation pathway. Biosci. Rep..

[41] Bellacosa A., Chan T.O., Ahmed N.N., Datta K., Malstrom S., Stokoe D., McCormick F., Feng J., Tsichlis P. (1998). Akt activation by growth factors is a multiple-step process: The role of the PH domain. Oncogene.

[42] Chautard E., Loubeau G., Tchirkov A., Chassagne J., Vermot-Desroches C., Morel L., Verrelle P. (2010). Akt signaling pathway: A target for radiosensitizing human malignant glioma. Neuro. Oncol..

[43] Zhang S., Gong Y., Wang H., Li Z., Huang Y., Fu X., Xiang P., Fan T.Y. (2021). AS602801 sensitizes glioma cells to temozolomide and vincristine by blocking gap junction communication between glioma cells and astrocytes. J. Cell. Mol. Med..

[44] Mantovani F., Collavin L., Del Sal G. (2019). Mutant p53 as a guardian of the cancer cell. Cell Death Differ..

[45] Fujii J., Homma T., Osaki T. (2022). Superoxide Radicals in the Execution of Cell Death. Antioxidants.

[46] Ryter S.W., Hong P.K., Hoetzel A., Park J.W., Nakahira K., Wang X., Choi A.M.K. (2007). Mechanisms of cell death in oxidative stress. Antioxid. Redox Signal.

[47] Kostaras E., Kaserer T., Lazaro G., Heuss S.F., Hussain A., Casado P., Hayes A., Yandim C., Palaskas N., Yu Y. (2020). A systematic molecular and pharmacologic evaluation of AKT inhibitors reveals new insight into their biological activity. Br. J. Cancer.

[48] Singh N., Miner A., Hennis L., Mittal S. (2021). Mechanisms of temozolomide resistance in glioblastoma—A comprehensive review. Cancer Drug Resist..

[49] Li H.F., Kim J.S., Waldman T. (2009). Radiation-induced Akt activation modulates radioresistance in human glioblastoma cells. Radiat. Oncol..

[50] Ueno H., Tomiyama A., Yamaguchi H., Uekita T., Shirakihara T., Nakashima K., Otani N., Wada K., Sakai R., Arai H. (2015). Augmentation of invadopodia formation in temozolomide-resistant or adopted glioma is regulated by c-Jun terminal kinase-paxillin axis. Biochem. Biophys. Res. Commun..

[51] Yu Z., Xie G., Zhou G., Cheng Y., Zhang G., Yao G., Chen Y., Li Y., Zhao G. (2015). NVP-BEZ235, a novel dual PI3K-mTOR inhibitor displays anti-glioma activity and reduces chemoresistance to temozolomide in human glioma cells. Cancer Lett..

[52] Munoz J.L., Rodriguez-Cruz V., Greco S.J., Ramkissoon S.H., Ligon K.L., Rameshwar P. (2014). Temozolomide resistance in glioblastoma cells occurs partly through epidermal growth factor receptor-mediated induction of connexin 43. Cell Death Dis..

[53] Lin X.M., Shi X.X., Xiong L., Nie J.H., Ye H.S., Du J.Z., Liu J. (2021). Construction of IL-13 Receptor α2-Targeting Resveratrol Nanoparticles against Glioblastoma Cells: Therapeutic Efficacy and Molecular Effects. Int. J. Mol. Sci..

[54] Galati S., Boni C., Gerra M.C., Lazzaretti M., Buschini A. (2019). Autophagy: A Player in response to Oxidative Stress and DNA Damage. Oxid. Med. Cell. Longev..

[55] Beck R., Verrax J., Dejeans N., Taper H., Calderon P.B. (2009). Menadione reduction by pharmacological doses of ascorbate induces an oxidative stress that kills breast cancer cells. Int. J. Toxicol..

[56] Osada S., Sakashita F., Hosono Y., Nonaka K., Tokuyama Y., Tanaka H., Sasaki Y., Tomita H., Komori S., Matsui S. (2008). Extracellular signal-regulated kinase phosphorylation due to menadione-induced arylation mediates growth inhibition of pancreas cancer cells. Cancer Chemother. Pharmacol..

[57] Adnan H., Antenos M., Kirby G.M. (2012). The effect of menadione on glutathione S-transferase A1 (GSTA1): C-Jun N-terminal kinase (JNK) complex dissociation in human colonic adenocarcinoma Caco-2 cells. Toxicol. Lett..

[58] Cao J., Xu D., Wang D., Wu R., Zhang L., Zhu H., He Q., Yang B. (2009). ROS-driven Akt dephosphorylation at Ser-473 is involved in 4-HPR-mediated apoptosis in NB4 cells. Free Radic. Biol. Med..

[59] Goetting I., Larafa S., Eul K., Kunin M., Jakob B., Matschke J., Jendrossek V. (2022). Targeting AKT-Dependent Regulation of Antioxidant Defense Sensitizes AKT-E17K Expressing Cancer Cells to Ionizing Radiation. Front. Oncol..

[60] Checa J., Aran J.M. (2020). Reactive Oxygen Species: Drivers of Physiological and Pathological Processes. J. Inflamm. Res..

[61] Shaukat Z., Liu D., Hussain R., Khan M., Gregory S.L. (2016). The Role of JNK Signalling in Responses to Oxidative DNA Damage. Curr. Drug Targets.

[62] Cheng S.Y., Chen N.F., Kuo H.M., Yang S.N., Sung C.S., Sung P.J., Wen Z.H., Chen W.F. (2018). Prodigiosin stimulates endoplasmic reticulum stress and induces autophagic cell death in glioblastoma cells. Apoptosis.

[63] López-Sánchez N., Rodríguez J.R., Frade J.M. (2007). Mitochondrial c-Jun NH2-terminal kinase prevents the accumulation of reactive oxygen species and reduces necrotic damage in neural tumor cells that lack trophic support. Mol. Cancer Res..

[64] Courtial L., Picco V., Grover R., Cormerais Y., Rottier C., Labbe A., Pagès G., Ferrier-Pagès C. (2017). The c-Jun N-terminal kinase prevents oxidative stress induced by UV and thermal stresses in corals and human cells. Sci. Rep..

[65] Amir M., Liu K., Zhao E., Czaja M.J. (2012). Distinct functions of JNK and c-Jun in oxidant-induced hepatocyte death. J. Cell. Biochem..

[66] Lamb J.A., Ventura J.J., Hess P., Flavell R.A., Davis R.J. (2003). JunD mediates survival signaling by the JNK signal transduction pathway. Mol. Cell..

[67] Qin L.-S., Yu Z.Q., Zhang S.M., Sun G., Zhu J., Xu J., Guo J., Fu L.S. (2013). =The short chain cell-permeable ceramide (C6) restores cell apoptosis and perifosine sensitivity in cultured glioblastoma cells. Mol. Biol. Rep..

[68] Cheng Y., Ren X., Zhang Y., Patel R., Sharma A., Wu H., Robertson G.P., Yan L., Rubin E., Yang J.M. (2011). eEF-2 kinase dictates cross-talk between autophagy and apoptosis induced by Akt Inhibition, thereby modulating cytotoxicity of novel Akt inhibitor MK-2206. Cancer Res..

[69] Feng F., Zhang M., Yang C., Heng X., Wu X. (2019). The dual roles of autophagy in gliomagenesis and clinical therapy strategies based on autophagic regulation mechanisms. Biomed. Pharmacother..

[70] Fulda S., Kögel D. (2015). Cell death by autophagy: Emerging molecular mechanisms and implications for cancer therapy. Oncogene.

[71] Vita M.F., Nagachar N., Avramidis D., Delwar Z.M., Cruz M.H., Siden Å., Paulsson K.M., Yakisich J.S. (2011). Pankiller effect of prolonged exposure to menadione on glioma cells: Potentiation by vitamin C. Invest. New Drugs.

[72] Benn C.L., Dawson L.A. (2020). Clinically Precedented Protein Kinases: Rationale for Their Use in Neurodegenerative Disease. Front. Aging Neurosci..

[73] Blahovcova E., Richterova R., Kolarovszki B., Dobrota D., Racay P., Hatok J. (2015). Apoptosis-related gene expression in tumor tissue samples obtained from patients diagnosed with glioblastoma multiforme. Int. J. Mol. Med..

