# Supplementary material for: Pharmacological Akt and JNK Kinase Inhibitors 10-DEBC and SP600125 Potentiate Anti-Glioblastoma Effect of Menadione and Ascorbic Acid Combination in Human U251 Glioblastoma Cells
Source: Biomedicines. 2023 Sep 27;11(10):2652. doi: 10.3390/biomedicines11102652 (PMC10604608; doi:10.3390/biomedicines11102652)
Supplement: Supplementary file 1 [file biomedicines-11-02652-s001.zip › biomedicines-2561622-supplementary.pdf]

Pharmacological Akt and JNK kinase inhibitors 10-DEBC and SP600125 potentiate anti-glioblastoma effect of menadione and ascorbic acid combination in human U251 glioblastoma cells

Ana Despotović, Kristina Janjetović, Nevena Zogović and Gordana Tovilović-Kovačević

Supplementary material

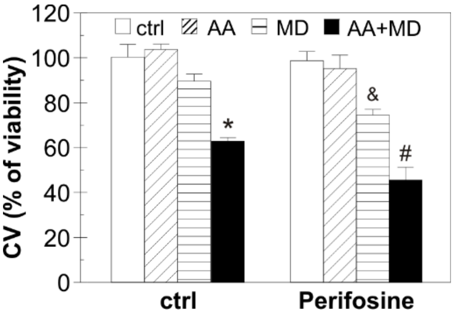

Figure S1

Figure S1. Akt inhibitor perifosine potentiates AA+MD-triggered U251 cell death and induces MD cytotoxicity. Untransfected U251 cells were pre-treated with perifosine (10 μM) and then exposed to AA (1 mM), MD (20 μM), and their combination (AA+MD) for 16h. Cell viability was assessed by crystal violet assay. The data are presented as mean ± SD values of triplicates from one representative of three independent experiments. *p<0.05 compared to control, untreated cells (ctrl), #p<0.05 compared to cells treated with AA+MD, &p<0.05 compared to cells treated with MD.

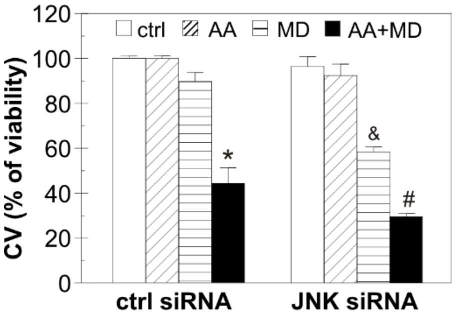

Figure S2

Figure S2. JNK downregulation with siRNA potentiates AA+MD-triggered U251 cell death and induces MD cytotoxicity. U251 cells transfected with control or JNK siRNA were treated with AA (1 mM), MD (20 μM), and their combination (AA+MD) for 16h. Cell viability was assessed by crystal violet assay. The data are presented as mean ± SD values of triplicates from one representative of three independent experiments. *p<0.05 compared to control, untreated cells (ctrl), #p<0.05 compared to control cells treated with AA+MD, &p<0.05 compared to control cells treated with MD.

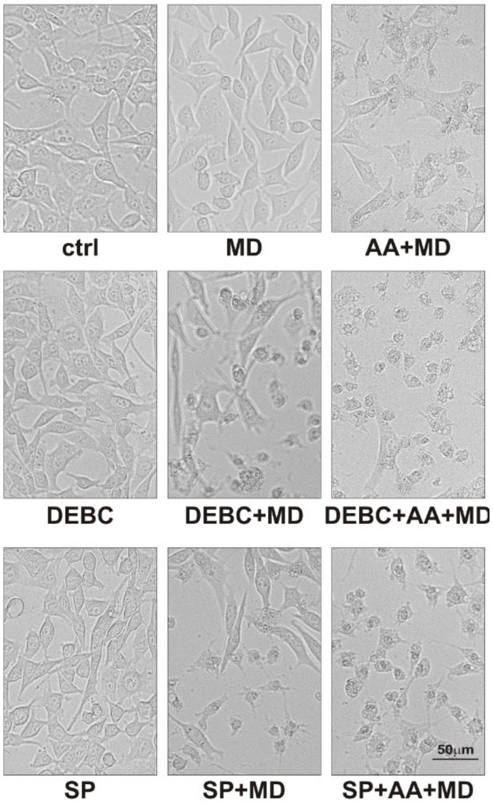

Figure S3

Figure S3. Morphological changes of U251 cells treated with 10-DEBC, SP, MD, and the combination. U251 cells were pre-treated with 10-DEBC (10 μM) and SP (10 μM) for 30 min and then treated with MD (20 μM) and AA+MD (1 mM+20 μM). The changes in morphology were observed after 6 hours of treatment using Bio-rad ZOE Cell Imager.

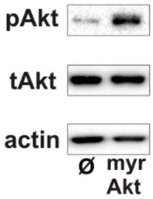

Figure S4

Figure S4. Myristoylated Akt transfection efficiency. U251 cells were transfected with PCDNA control construct (1 ng/μl) and Myr-Akt (1 ng/μl). Cells were allowed to grow 24h following transfection and immunoblot analysis of phospho-Akt (pAkt), total-Akt (tAkt), and actin was performed to confirm transfection efficiency. Representative immunoblots are shown.
